# Methotrexate-Induced Mucositis: A Consequence of Medication Error in a Rheumatoid Arthritis Patient

**DOI:** 10.7759/cureus.46290

**Published:** 2023-09-30

**Authors:** Rujen Shrestha, Shiva K Ojha, Suman Kumar Jha, Ranjit Jasraj, Abhishek Fauzdar

**Affiliations:** 1 Internal Medicine, Scheer Memorial Adventist Hospital, Banepa, NPL; 2 Family Medicine, University of North Dakota, Fargo, USA; 3 Internal Medicine, Mount Sinai Medical Center, Chicago, USA

**Keywords:** rheumatoid arthritis, folinic acid, mucositis, medication error, methotrexate

## Abstract

Methotrexate (MTX) is a commonly prescribed medication for the treatment of rheumatoid arthritis (RA). Although effective in managing rheumatoid arthritis symptoms, methotrexate can have adverse effects, including mucositis. This study highlights a case of methotrexate-induced mucositis resulting from a medication error in a patient with rheumatoid arthritis. The 69-year-old patient recently diagnosed with rheumatoid arthritis was receiving methotrexate therapy as a part of his treatment plan. The patient, however, unintentionally ingested an excessive dose of methotrexate because of a communication error that occurred during the medication administration process. He started displaying signs of oral mucositis, characterized by uncomfortable ulcers and oral mucosa inflammation, within a short period. The buccal mucosa, tongue, and gingiva of the patient's oral cavity displayed numerous ulcerative lesions upon examination. Due to the mucositis's severity, it was challenging to eat, speak, and perform regular oral hygiene procedures. The patient described severe discomfort that had a detrimental effect on his general quality of life. This case serves as a reminder of the importance of accurate medication administration and communication in the management of rheumatoid arthritis. Healthcare professionals should ensure proper dosing and monitoring to minimize the risk of medication errors and associated complications. Additionally, patients should be educated about the potential side effects of methotrexate, including mucositis, to enable early recognition and timely intervention. In conclusion, this study emphasizes the occurrence of methotrexate-induced mucositis because of medication administration errors in a patient with rheumatoid arthritis. By increasing awareness of this potential complication, healthcare providers can improve patient safety and enhance the overall management of rheumatoid arthritis treatment.

## Introduction

A folic acid antagonist, methotrexate (4-amino-N10-methyl-pteroylglutamic acid) is also known as aminopterin (4-amino-pteroylglutamic acid). It is currently unclear how methotrexate at low doses controls rheumatoid arthritis inflammation. Rapid clinical remission following methotrexate withdrawal indicates that the anti-inflammatory components of methotrexate mechanisms of action are far more important for treating rheumatoid arthritis than the anti-proliferative ones [1]. Methotrexate has been used in the treatment of rheumatoid arthritis (RA) since the 1980s and to this day is often the first-line medication for rheumatoid arthritis treatment [2]. A dose of 5-35 mg methotrexate is typically administered once per week. Toxicities to the bone marrow and digestive systems are the most frequent side effects of methotrexate therapy. Additionally, these side effects are dose-dependent and potentially fatal; as a result, when they appear, methotrexate treatment is reduced or discontinued, and folic acid is given. Although stomatitis is a frequent side effect of methotrexate therapy when treating malignant disease, it is uncommon when using methotrexate therapy to treat rheumatoid arthritis [3]. By substituting folate or folinic acid (leucovorin), methotrexate toxicity can be treated and reversed if timely intervention is done [4]. Despite being treated with methotrexate, leucovorin, the reduced form of folate, can restore the cell's capacity to manufacture deoxyribonucleic acid (DNA) bases because it avoids the dihydrofolate reductase enzyme, which is the target of methotrexate [5].

## Case presentation

**Figure 1:**
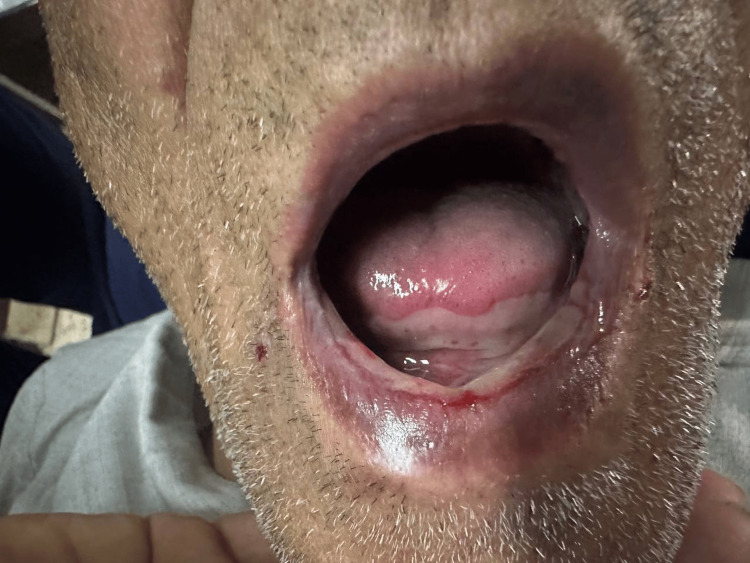
Dry lips with concomitant cheilitis.

**Figure 2:**
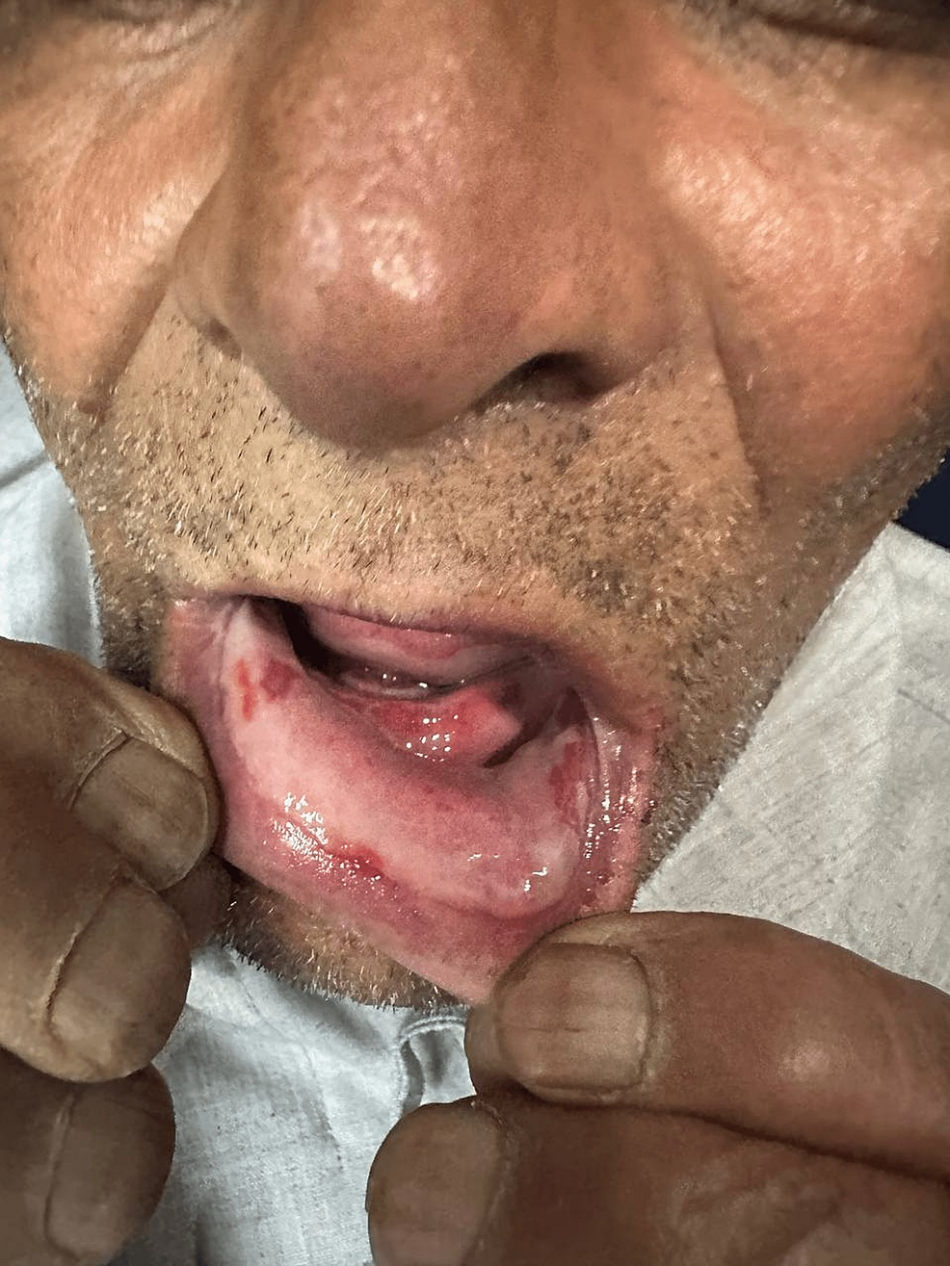
Erythematous and ulcerative buccal mucosa.

A 69-year-old male with rheumatoid arthritis (RA) presented to the emergency department with a chief complaint of dysphagia for the last five days. Clinical examination showed many ulcers in the buccal mucosa, tongue, and lips (Figure 1) and an inflamed painful oral cavity which is consistent with WHO grade 4 oral mucositis (Figure 2) [6]. His rheumatoid Arthritis was being treated with non-steroidal anti-inflammatory drugs (aceclofenac 100 mg twice daily).

He did not have any significant past medical history. He had lately been administered methotrexate 7.5 mg orally once weekly to treat his rheumatoid arthritis. However, the patient had misjudged the two medications and taken methotrexate 7.5 mg twice daily for the past five days and started developing symptoms. His history and diagnostic findings were consistent with a methotrexate overdose that caused this acute reaction. The patient was admitted to the hospital for the management of potentially fatal bone marrow suppression due to his generalized impairment, dehydration, and inability to tolerate oral feeding. Upon hospitalization, he was afebrile, and his vital signs showed a blood pressure of 110/70 mmHg, a respiratory rate of 22 breaths per min, and a pulse of 84 beats per min.

His initial laboratory blood testing showed early signs of bone marrow suppression (white blood count: 2900 cells/cumm, platelets: 147,000 cells/cumm) and elevated liver enzymes showing signs of transaminitis (aspartate aminotransferase {AST}: 70 U/L, alanine aminotransferase {ALT}: 55 U/L). Urine routine analysis and renal function test were normal at the time of presentation. Parenteral rehydration was initiated, antiemetics (intravenous ondansetron), and analgesics (ointment triamcinolone acetonide, diclofenac mouthwash) were given for comfort, and methotrexate was discontinued. Leucovorin was started via intravenous route (IV) with a dose of 15 mg every 6 hours for a total of 10 doses. Blood sugar was monitored every 8 hours. The patient was kept isolated because of an immunocompromised state. On day third, the patient developed features of a urinary tract infection, and intravenous antibiotics (ceftriaxone 2 g once daily) were started. The patient was complaining of persistent oral pain so analgesics were changed to ointment Zytee (choline salicylate + benzalkonium chloride) and ointment Quadragel (lidocaine + chlorhexidine gluconate + metronidazole) prescribed. The patient's symptoms started improving. Oral nutrition was resumed after two days. The patient remained hospitalized for six days, and blood counts (platelets: 125,000 cells/cumm) were improving and liver function (AST: 46 U/L, ALT: 55 U/L) recovered, as confirmed by laboratory tests on day three of hospitalization (Table 1).

**Table 1:** Results of laboratory tests during the patient’s hospitalization. AST: aspartate aminotransferase; ALT: alanine transaminase

Test	Day 1	Day 2	Day 3	Day 4	Day 5	Day 6	Day 7	Normal
White blood cells (cells/cumm)	2900	3800	1700	2300	2300	2300	2800	3500-11,500
Hemoglobin (mg/dL)	12.1	11.3	10.5	12.1	11.7	12.1	12	12-18
Platelets (cells/cumm)	147,000	126,000	95,000	110,000	90,000	91,000	125,000	150,000-410,000
AST (U/L)	70	-	48	-	-	-	-	0-50
ALT (U/L)	55	-	32	-	-	-	-	0-50
Urine pus cell (/hpf)	4-6	-	3-4	7-8	-	18-20	-	-

**Figure 3:**
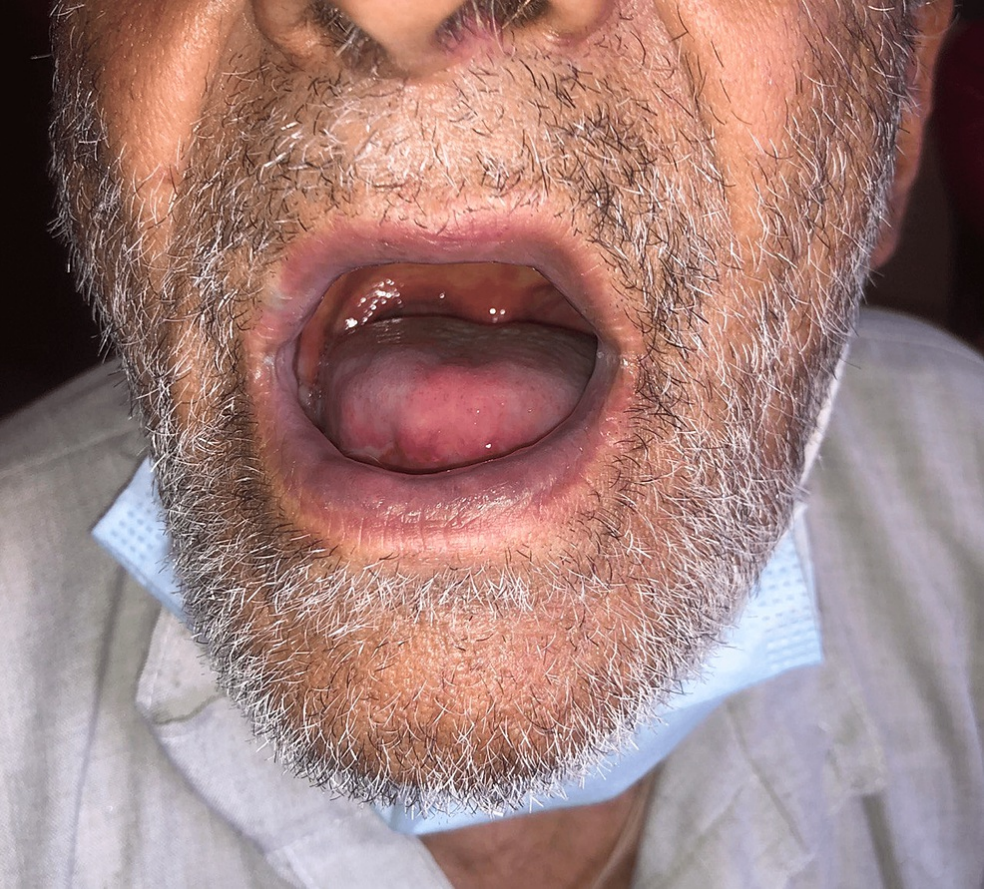
Healing lips ulcer and cheilitis.

**Figure 4:**
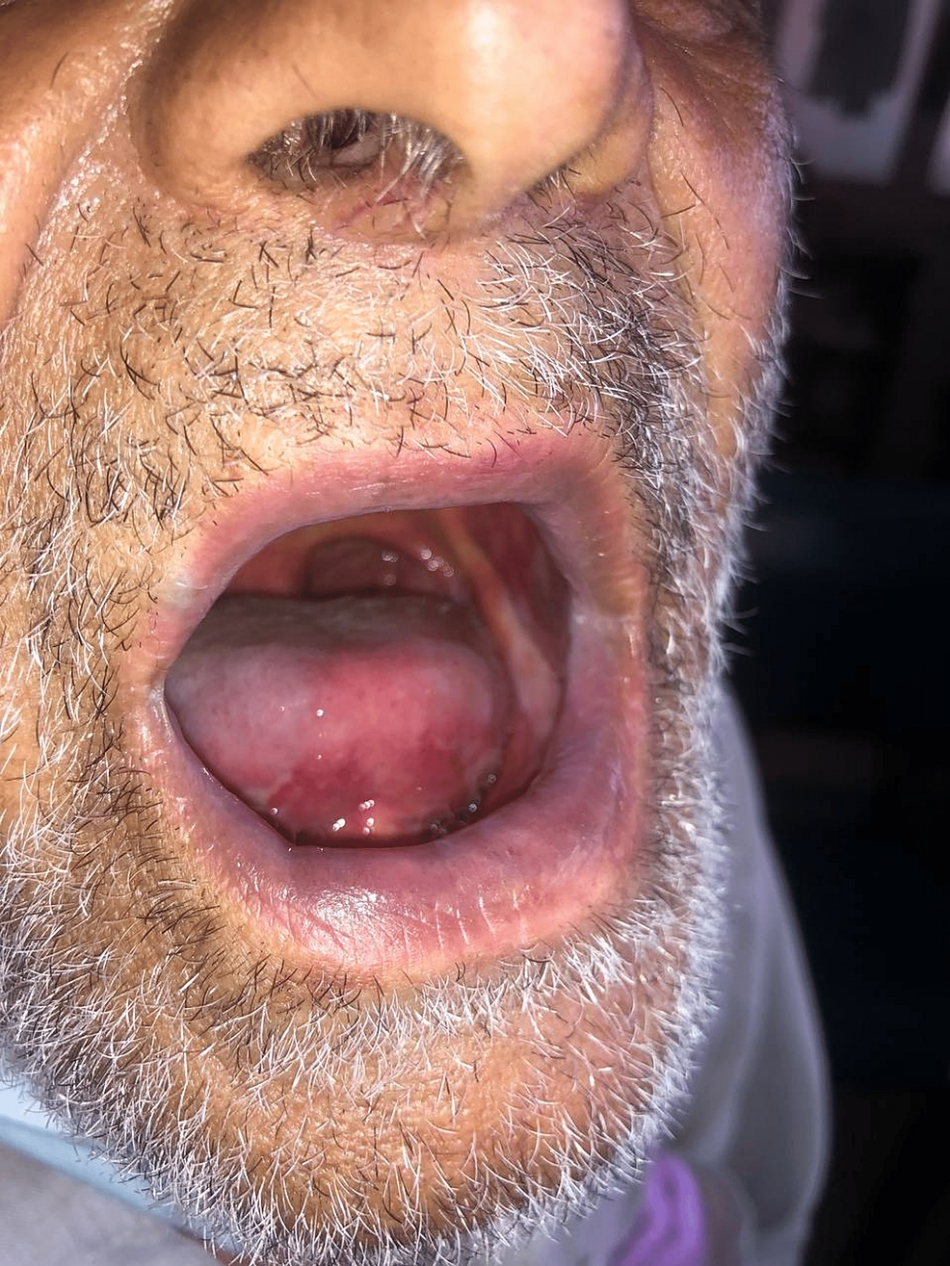
Healing buccal mucosa on the left side.

**Figure 5:**
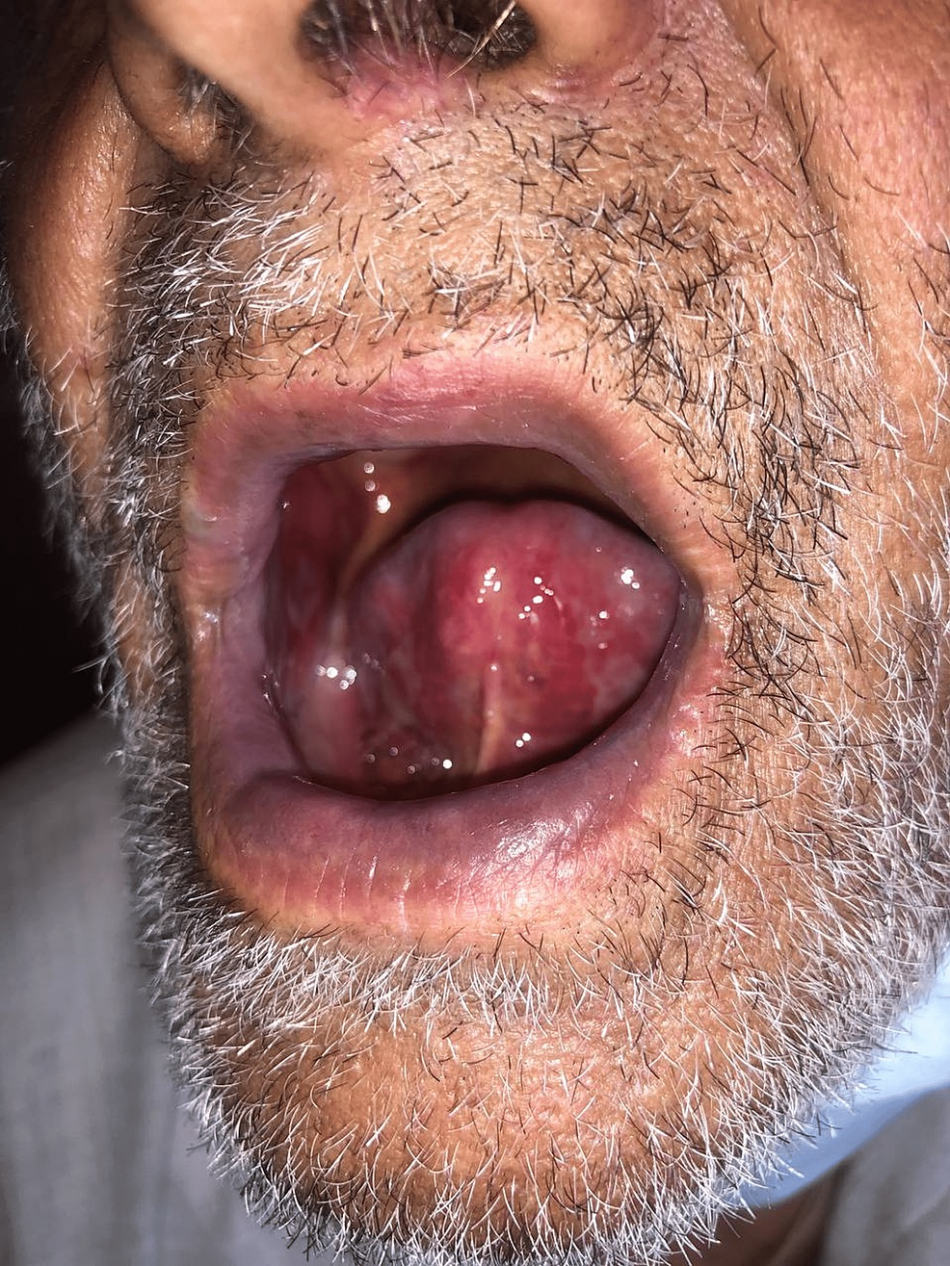
Healing buccal mucosa on the right side.

The patient had been discharged with better overall health, no pain, and healing ulcerations on the skin and in the mouth; all ulcerations were resolved by a follow-up session one week later. The patient showed a healed lip ulcer with the absence of cheilitis which can be seen in Figure 3. Buccal mucosa of both sides showed significant improvement as displayed in Figures 4, 5. The patient was advised to have his next follow-up after two weeks.

## Discussion

Methotrexate is used to treat a variety of malignancies, autoimmune disorders, and abortions. Active cellular uptake and active efflux transporters allow it into the cells. Once methotrexate enters the cell, it inhibits the enzyme dihydrofolate reductase (DHFR), which converts dihydrofolate (DHF) into tetrahydrofolate (THF). Thymidylate and purine synthesis are consequently reduced. When DNA synthesis stops, cells eventually lose the ability to proliferate [7]. Because the medication is polyglutaminated, its intracellular presence is prolonged. As a result, cells, including lymphoblasts, synovial macrophages, and epithelia that are capable of effective polyglutamination, are more sensitive to this medication [8]. Misadministration of the medication can lead to serious complications, and, importantly, even at low doses, methotrexate toxicity has the potential to be fatal. The fast cellular turnover of these organs, where many cells are in the synthesis phase of the cell cycle and consequently particularly sensitive to the cytocidal effects of methotrexate, may explain why relatively low doses of methotrexate can elicit both cutaneous and hematologic damage [9].

The cornerstone of high-dose methotrexate toxicity management is leucovorin (folinic acid) rescue therapy. It challenges methotrexate for cell entry and enables intracellular folate replenishment [10]. Leucovorin rescue therapy should be started for those who develop pancytopenia and hematologic side effects as a result of low-dose methotrexate toxicity, either with or without granulocyte-colony stimulating factor (G-CSF) therapy. Additionally, the patient in the present case received 10 doses of leucovorin rescue therapy every six hours. By the fourteenth day after admission, the patient was responding to treatment and his pancytopenia was resolving.

The case highlights the importance of proper instruction for the consumption of medication for both the patient and the caregiver. Despite early detection of a medication error, the patient still developed pancytopenia, leading to a urinary tract infection. The National Coordinating Council for Medication Error Reporting and Prevention defines a medication error as any event that may be avoided that could result in improper drug usage or patient injury while the patient, consumer, or healthcare professional remains in charge of the medication. Such events may be linked to medical practice, healthcare items, methods, and systems, such as prescription writing, order communication, product labeling, packaging, and nomenclature, compounding, dispensing, distribution, administration, education, monitoring, and use [11]. Monitoring plasma methotrexate levels is crucial for avoiding the side effects of high-dose methotrexate therapy. Frequently, plasma methotrexate levels are measured 24 hours, 48 hours, and 72 hours after the start of a methotrexate infusion [12]. Our institute lacked the facility to obtain plasma methotrexate levels, so most of the prognosis for patients was based on clinical and other laboratory findings. As the kidneys are the primary organs responsible for the majority of methotrexate excretion, it is essential to evaluate renal function before, during, and after each cycle of medication. This involves measuring serum creatinine, blood urea nitrogen (BUN), urine output, and urine pH. Complete blood count, liver function test, renal function test, and chest radiograph should all be performed before commencing medication. Complete blood count (CBC), liver function test (LFT), and renal function test (RFT) should be assessed every two to four weeks for the first three months, every 8-12 weeks for the following three to six months, and then every 12 weeks after that [13].

## Conclusions

Methotrexate has a high prevalence of serious, avoidable medication mistakes, which calls for rigorous steps to increase patient safety. Patients must be fully informed about the dosage for each tablet, the total dose, the route, and the day of administration for folic acid and methotrexate. Patients should be warned to look out for any therapy-related problems. This gives the patient the chance to study any instructions that might deviate from standard practice. Caregivers of patients should be included in discussions as well. To reduce errors, they might be taught the proper amount and frequency of medicine delivery. Patients should be urged to adhere to the plan, and periodic blood test monitoring should be made available.
